# Serum Selenium Concentrations and Diabetes in U.S. Adults: National Health and Nutrition Examination Survey (NHANES) 2003–2004

**DOI:** 10.1289/ehp.0900704

**Published:** 2009-05-15

**Authors:** Martin Laclaustra, Ana Navas-Acien, Saverio Stranges, Jose M. Ordovas, Eliseo Guallar

**Affiliations:** 1 Department of Cardiovascular Epidemiology and Population Genetics, Centro Nacional de Investigaciones Cardiovasculares, Madrid, Spain; 2 Department of Epidemiology and Welch Center for Prevention, Epidemiology, and Clinical Research and; 3 Department of Environmental Health Sciences, Johns Hopkins Bloomberg School of Public Health, Baltimore, Maryland, USA; 4 Clinical Sciences Research Institute, Warwick Medical School, University Hospital Coventry and Warwickshire, Coventry, United Kingdom; 5 Nutrition and Genomics Laboratory, Jean Mayer–U.S. Department of Agriculture Human Nutrition Research Center on Aging, Tufts University, Boston, Massachusetts, USA

**Keywords:** diabetes, glycosylated hemoglobin, National Health and Nutrition Examination Survey, NHANES, selenium

## Abstract

**Background:**

Increasing evidence suggests that high selenium levels are associated with diabetes and other cardiometabolic risk factors.

**Objectives:**

We evaluated the association of serum selenium concentrations with fasting plasma glucose, glycosylated hemoglobin levels, and diabetes in the most recently available representative sample of the U.S. population.

**Methods:**

We used a cross-sectional analysis of 917 adults ≥ 40 years of age who had a fasting morning blood sample in the National Health and Nutrition Examination Survey 2003–2004. We evaluated the association of serum selenium, measured by inductively coupled plasma-dynamic reaction cell-mass spectrometry, and diabetes, defined as a self-report of current use of hypoglycemic agents or insulin or as fasting plasma glucose ≥ 126 mg/dL.

**Results:**

Mean serum selenium was 137.1 μg/L. The multivariable adjusted odds ratio [95% confidence interval (CI)] for diabetes comparing the highest quartile of serum selenium (≥ 147 μg/L) with the lowest (< 124 μg/L) was 7.64 (3.34–17.46). The corresponding average differences (95% CI) in fasting plasma glucose and glycosylated hemoglobin were 9.5 mg/dL (3.4–15.6 mg/dL) and 0.30% (0.14–0.46%), respectively. In spline regression models, the prevalence of diabetes as well as glucose and glycosylated hemoglobin levels increased with increasing selenium concentrations up to 160 μg/L.

**Conclusions:**

In U.S. adults, high serum selenium concentrations were associated with higher prevalence of diabetes and higher fasting plasma glucose and glycosylated hemoglobin levels. Given high selenium intake in the U.S. population, further research is needed to determine the role of excess selenium levels in the development or the progression of diabetes.

Most Americans have selenium intake ranging from 60 to 220 μg/day (Combs 2001), well above the recommended dietary allowance of 55 μg/day (Institute of Medicine and Panel on Dietary Antioxidants and Related Compounds 2000; Rayman 2008). This high level of intake, particularly compared with other countries, is attributable to the high soil content of selenium in several areas of the United States, which is eventually incorporated in the food chain (Rayman 2008). Although selenium is required for adequate function of glutathione peroxidase and other selenoproteins, the risk of selenium deficiency in the U.S. general population is negligible. Additional selenium intake at high intake levels does not increase glutathione peroxidase synthesis or activity, but rather increases plasma selenium concentration by the non-specific incorporation of selenomethionine into plasma proteins (Institute of Medicine and Panel on Dietary Antioxidants and Related Compounds 2000), with unknown health effects.

Bleys et al. (2007) reported a positive association between serum selenium concentrations and the prevalence of diabetes among participants in the Third National Health and Nutrition Examination Survey (NHANES III). Stranges et al. (2007) reported an increased risk of diabetes among participants randomized to long-term selenium supplementation (200 μg/day) in the Nutritional Prevention of Cancer (NPC) trial. Recently, the Selenium and Vitamin E Cancer Prevention Trial (SELECT), a mega-trial aimed to evaluate the efficacy of selenium in preventing prostate cancer, was prematurely stopped by the data and safety monitoring committee because of lack of benefit on the primary end point and the possibility of an increased risk of diabetes in the selenium-only arm (Lippman et al. 2009). Additionally, high selenium status has been linked with hypercholesterolemia (Bleys et al. 2008b) and hypertension (Laclaustra et al. 2009) in the U.S. population.

Because there is a narrow range between selenium intake levels required for selenoprotein synthesis and toxic levels (Institute of Medicine and Panel on Dietary Antioxidants and Related Compounds 2000), these findings raise concerns of possible adverse cardio-metabolic effects of high selenium exposure, at least in selenium-replete populations such as the United States. NHANES III and the NPC trial, however, were conducted during the 1980s and early 1990s, and there are no recent data on the association of serum selenium levels with diabetes in the United States. The objective of this study was to evaluate the association of serum selenium concentrations with the prevalence of diabetes using recently available NHANES data collected in 2003–2004. Moreover, we also evaluated the association between serum selenium levels with fasting plasma glucose and glycosylated hemoglobin levels, biomarkers not previously considered.

## Materials and Methods

NHANES is conducted by the National Center for Health Statistics (NCHS) using a complex multistage sampling design to obtain a probability sample of the civilian non-institutionalized U.S. population. We used data from NHANES 2003–2004 (NCHS 2003–2004), the most recent release with selenium data available in adults. Serum selenium measurements were restricted to participants ≥ 40 years of age (*n* = 3,299), of whom 1,302 participated in the morning examination and had a fasting blood sample. Among these, 1,273 participants had serum selenium measurements. To minimize the possibility of reverse causation in the associations examined, we excluded participants with self-reported coronary heart disease (*n* = 156), stroke (*n* = 80), or cancer (*n* = 173), leaving 934 participants. We further excluded 16 participants with missing body mass index (BMI) and 1 participant missing cotinine concentration. The final sample size was 917.

### Serum selenium

Collection materials were screened for potential selenium contamination. Serum selenium was measured at the Trace Elements Laboratory at the Wadsworth Center of the New York State Department of Health (Albany, NY, USA) using inductively coupled plasma–dynamic reaction cell–mass spectrometry. The between-assay coefficients of variation for quality control pooled samples analyzed throughout the duration of the survey ranged from 2.5% to 2.9% (NCHS 2003–2004).

### Glucose, glycosylated hemoglobin, and diabetes

Plasma glucose was measured from a morning fasting sample of participants who fasted 8–24 hr by the enzyme hexokinase method (NCHS 2003–2004). Diabetes was defined as the presence of either a self-report of current use of hypoglycemic agents or insulin or fasting plasma glucose ≥ 126 mg/dL. Similar associations between selenium and diabetes were found if the definition of diabetes was based only on questionnaire or only on fasting plasma glucose levels (data not shown).

Glycosylated hemoglobin measurements were performed using the Primus CLC330 and Primus CLC385 boronate affinity high-performance liquid chromatography systems (Primus Corp., Kansas City, MO, USA). The systems were standardized to the reference method used for the Diabetes Control and Complications Trial (DCCT; 1993). The long-term interassay coefficient of variation was < 3.0% (NCHS 2003–2004).

### Other variables

Information on sex, age, race/ethnicity, education, menopausal status, smoking, and use of vitamin/mineral supplements (as a single yes/no question, including those supplements containing selenium) was based on self-report. BMI was calculated by dividing measured weight in kilograms by measured height in meters squared. Serum cotinine was measured by isotope-dilution high-performance liquid chromatography/atmospheric pressure chemical ionization tandem mass spectrometry (NCHS 2003–2004).

### Statistical methods

Participants were divided into quartiles of serum selenium concentration based on the weighted population distribution. We used multivariable linear regression to calculate adjusted means for plasma glucose and glycosylated hemoglobin differences and logistic regression to calculate odds ratios (ORs) for diabetes comparing each quartile of serum selenium with the lowest quartile. We used three models with progressive degrees of adjustment. Model 1 was adjusted for sex, age, race/ethnicity, and education. Model 2 was further adjusted for BMI, smoking, cotinine, and menopausal status. Model 3 was further adjusted for use of vitamin/mineral supplements. Because ignoring diabetes treatment may result in biased estimates of the association between selenium and glucose or glycosylated hemoglobin, we conducted an additional analysis using censored linear regression (model 4) to correct for the effect of medication for diabetes using NHANES survey weights (Tobin et al. 2005). Tests for linear trend were calculated by including serum selenium as a continuous variable in the models. To further explore the shape of the relationship among serum selenium and plasma glucose, glycosylated hemoglobin, and diabetes, we used restricted quadratic splines with knots at the 5th, 50th, and 95th percentiles of the serum selenium distribution. We also evaluated the interactions between selenium (modeled as quadratic restricted splines) and sex, age, race/ethnicity, education, BMI, smoking status, or use of vitamin/mineral supplements. Statistical analyses were performed using weights specific for the fasting morning sample in the survey package in the R Statistical Software (version 2.6.1; R Foundation for Statistical Computing, Vienna, Austria) to account for the complex sampling design and weights. Censored regression models were estimated using the cnreg command in Stata Statistical Software (release 9.2; StataCorp LP, College Station, TX, USA) weighted for NHANES survey weights.

## Results

The mean (± SD) serum selenium concentration in the study population was 137.1 ± 19.9 μg/L. The overall prevalence of diabetes was 10.0%. Participants with diabetes were more likely to be older, male, and non-Hispanic black or Mexican American and to have a higher BMI compared with participants without diabetes (Table 1). Serum selenium concentrations were positively associated with age and with the use of vitamin/mineral supplements, and inversely associated with current smoking (Table 2). Men had higher mean serum selenium than did women (138.6 vs. 135.9 μg/L). Non-Hispanic blacks had lower mean serum selenium (129.0 μg/L) compared with non-Hispanic whites (138.2 μg/L) and with Mexican Americans (140.4 μg/L).

**Figure 1:**
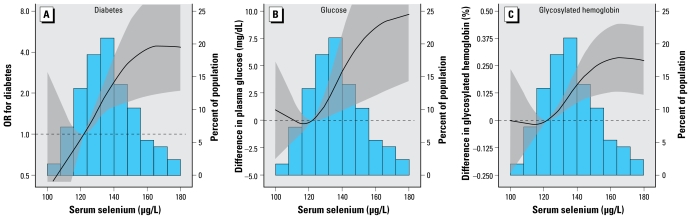
Adjusted ORs (curves) and 95% CIs (gray shading) for diabetes (*A*) and adjusted differences (and 95% CI) in fasting glucose (*B*) and glycosylated hemoglobin (*C*) by serum selenium concentration. Serum selenium was modeled as restricted quadratic splines with nodes at the 5th, 50th, and 95th percentiles. The multivariable linear regression models were adjusted for sex, age, race, education, BMI, smoking, cotinine, postmenopausal status, and use of vitamin and mineral supplements (model 3). The odds for diabetes and the values of the continuous variables at the 20th percentile (122 μg/L) of the serum selenium distribution were used as reference. The histogram shows the distribution of selenium concentrations in the study population.

**Figure 2:**
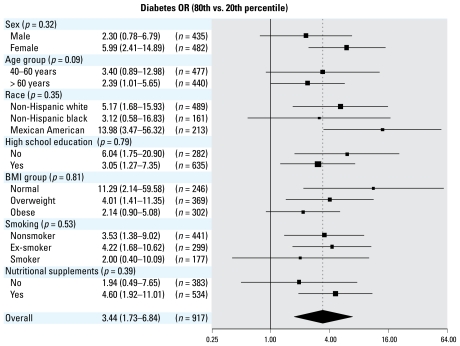
Adjusted ORs (95% CIs) for diabetes comparing the 80th percentile (150 μg/L) with the 20th percentile (122 μg/L) of the serum selenium distribution. Serum selenium was modeled as restricted quadratic splines with nodes at the 5th, 50th, and 95th percentiles. Multivariable logistic regression models were adjusted for sex, age, race, education, BMI, smoking, cotinine, postmenopausal status, and use of vitamin and mineral supplements (model 3). The size of the square indicates the number of participants in each stratum. *p*-Values correspond to tests for interaction between selenium splines and selected participant characteristics.

Mean serum selenium concentrations were higher in participants with diabetes compared with those without it (143.7 vs. 136.4 μg/L, *p* = 0.001). The multivariable adjusted OR [95% confidence interval (CI)] for diabetes comparing the highest selenium quartile (≥ 147 μg/L) with the lowest (< 124 μg/L) was 7.64 (95% CI, 3.34–17.46) (Table 3, model 3). In spline regression models, the prevalence of diabetes increased with increasing selenium concentrations up to 160 μg/L (Figure 1, left). Adjusted ORs for diabetes comparing the 80th percentile (150 μg/L) with the 20th (122 μg/L) percentile of the selenium distribution showed consistent findings across clinically relevant subgroups (Figure 2).

In multivariable adjusted models, the average differences (95% CI) comparing the highest with the lowest selenium quartile were 9.5 mg/dL (3.4–15.6 mg/dL) for fasting plasma glucose, and 0.30% (0.14–0.46%) for glycosylated hemoglobin (Table 3, model 3). In spline regression models, levels of both variables increased with increasing selenium concentrations up to 160 μg/L (Figure 1, center and right). Extending the definition of diabetes to include all participants with a previous doctor’s diagnosis of diabetes (including nontreated participants) or further adjustment for waist circumference and family history of diabetes produced similar results (data not shown).

## Discussion

In this representative cross-sectional study of the U.S. population conducted in 2003–2004, high serum selenium concentrations were associated with a higher prevalence of diabetes, as well as with higher fasting plasma glucose and glycosylated hemoglobin levels. These data are consistent with the previous observation of an increased risk of diabetes with high selenium concentrations in NHANES III, conducted in 1988–1994 (Bleys et al. 2007). From 1988–1994 to 2003–2004, however, serum selenium concentrations among U.S. subjects ≥ 40 years of age increased from 125.8 μg/L (95% CI, 124.1–127.6 μg/L) in NHANES III (*n* = 9,085) to 136.6 μg/L (134.4–138.7 μg/L) in NHANES 2003–2004 (*n* = 2,903). This increasing trend in serum selenium levels highlights the importance of understanding the association between serum selenium levels and diabetes. Moreover, the NPC trial—a randomized, double-blind clinical trial to evaluate the efficacy of selenium supplementation (200 μg/day) for the prevention of cancer—showed an increased risk of diabetes after 7.7 years of follow-up (hazard ratio, 1.50; 95% CI, 1.03–2.33) comparing selenium supplementation with placebo (Stranges et al. 2007). Interestingly, the excess risk of diabetes with selenium supplementation in the NPC trial was restricted to participants in the highest tertile of plasma selenium at baseline (> 121.6 μg/L). More recently, SELECT, a large randomized clinical trial to evaluate the efficacy of selenium supplements (200 μg/day) on prostate cancer prevention, found more cases of diabetes in the group taking only selenium compared with placebo (relative risk, 1.07; 99% CI, 0.94–1.22; *p* = 0.16) but not in the selenium plus vitamin E group (relative risk, 0.97; 99% CI, 0.85–1.11; *p* = 0.62) (Lippman et al. 2009).

Our findings are consistent with recent reports from several U.S. studies of positive associations of selenium concentrations with lipid levels (Bleys et al. 2008b) and with hypertension (Laclaustra et al. 2009), as well as of U-shaped relationships with cardiovascular end points (Bleys et al. 2008a, 2009; Flores-Mateo et al. 2006; Salvini et al. 1995). Given the high selenium exposure in the United States, the mechanisms underlying these associations need to be investigated.

The high soil content of selenium in several areas of the United States and the use of supplements (Rayman 2008) explain the high mean selenium concentration in our sample (137 μg/L). This level is high compared with selenium needs for optimizing selenoprotein synthesis and activity (Institute of Medicine and Panel on Dietary Antioxidants and Related Compounds 2000) and relative to selenium levels in other populations (Rayman 2008). Glutathione peroxidases synthesis plateaus above serum selenium levels of 70–90 μg/L (Combs 2001). Above this threshold, serum selenium levels do not correlate with glutathione peroxidase activity or with mRNA synthesis for a variety of selenoproteins, but reflect variation in selenomethionine that is incorporated nonspecifically in serum proteins, mainly in albumin (Institute of Medicine and Panel on Dietary Antioxidants and Related Compounds 2000), with unknown physiologic activity. Because only one participant in our sample had serum selenium levels < 90 μg/L, variability in serum selenium levels is unlikely to reflect underlying variation in selenoprotein levels.

Many other countries, including most of Europe, have substantially lower serum selenium levels (mean values < 100 μg/L) (Rayman 2000), mainly due to poorer soil content, so our findings may not extrapolate to them. In an observational analysis of the Supplementation with Antioxidant Vitamins and Minerals trial (SU.VI.MAX) conducted in France, plasma selenium concentration at baseline (mean, 87 μg/L) was positively associated with fasting plasma glucose at baseline and after 7.5 years of follow-up (Czernichow et al. 2006). However, no differences in fasting glucose levels were found in the randomized component of the trial, comparing a multivitamin supplement that included 100 μg/day of selenium versus placebo over the follow-up (Czernichow et al. 2006). In a cross-sectional analysis of the French Etude du Viellissement Artérial (EVA), Coudray et al. (1997) found no statistically significant correlations between plasma selenium (mean, 87 μg/L) and fasting plasma glucose or diabetes prevalence. Finally, a small cross-sectional study of Asian persons residing in Singapore also found similar mean serum selenium levels among participants with and without diabetes (Hughes et al. 1998).

An excess of reactive oxygen species may increase insulin resistance and affect pancreatic β-cell function (Houstis et al. 2006), and some selenium compounds such as selenite and methylselenol (a metabolite of selenomethionine) (Rayman 2005) can induce oxidative stress (Drake 2006; Spallholz 1994; Spallholz et al. 2004). However, whereas substantial attention has been paid to explain the mechanisms for a potential benefit of increasing serum selenium in low-selenium intake populations, the mechanistic explanation for the effects of selenium above the levels required to maximize glutathione peroxidase activity is largely unexplored. Further experimental and epidemiologic research is needed to explain the mechanisms underlying the observed associations between high selenium exposure and cardiometabolic risk factors.

Higher selenium concentrations were associated with some diabetes risk factors such as increasing age. In contrast, selenium concentrations decreased with higher BMI, which is one of the stronger diabetes risk factors. The associations of selenium and diabetes reported were adjusted for these risk factors, reducing the possibility that the association between selenium and diabetes is driven by differences in established diabetes risk factors across selenium levels.

The strengths of our study come from the rigorous sampling design, the quality of the study measurements, and the representativeness of the NHANES sample. Among its limitations, the cross-sectional design restricts our ability to evaluate the temporality of the observed association. It is possible that high serum selenium levels are related to body weight. Nevertheless, the observed association persisted after adjusting for BMI as well as for use of multivitamin/mineral supplements. The use of a single measurement of serum selenium may not reflect completely the known high within-person variability (Longnecker et al. 1993) and does not provide information on selenium metabolism. A cross-sectional analysis of a subsample of the Health Professionals Follow-up Study found an inverse association between toenail selenium levels and the prevalence of diabetes (Rajpathak et al. 2005). Serum and toenail selenium are both biomarkers of selenium intake but are likely to reflect different selenium compartments. Toenail selenium could represent a longer trend of selenium intake than serum selenium, but there are no data supporting the grounds of their differences. More detailed analysis of different selenoproteins and related activities are needed to better understand the association of selenium with diabetes.

## Conclusions

In summary, high serum selenium concentrations were associated with higher prevalence of diabetes in a representative survey of U.S. adults conducted in 2003–2004. These findings are consistent with an earlier NHANES survey conducted in 1988–1994. Furthermore, these findings are supported by randomized evidence from the NPC trial and SELECT indicating that selenium supplementation may increase the risk of diabetes in selenium-replete populations such as that of the United States. High selenium concentrations have also been linked to increased lipid levels (Bleys et al. 2008b) and hypertension (Laclaustra et al. 2009). Given the current diabetes epidemic, the high selenium intake from naturally occurring selenium in U.S. soil, and the popularity of multivitamin/mineral supplements containing selenium in the United States, these findings call for a thorough evaluation of the risks and benefits associated with high selenium status in the United States. Furthermore, our findings suggest that selenium supplements should not be used in the United States until there is a better understanding of their potential risks and benefits.

## Figures and Tables

**Table 1:** Characteristics of the study population by diabetes status.

Characteristic	Overall	Normal	Diabetes	*p*-Value
No.	917	796 (90.0%)	121 (10.0%)	
Age (years)	54.2 ± 11.3	53.6 ± 11.1	59.4 ± 11.6	0.002
Sex (% female)	53.5	54.6	43.0	0.04
Race (%)				0.09
Non-Hispanic white	75.8	76.8	66.3	
Non-Hispanic black	10.2	9.8	13.8	
Mexican American	6.1	5.6	10.2	
Other	8.0	7.8	9.7	
Education (% high school)	83.8	84.4	77.7	0.11
BMI (kg/m^2^)	28.6 ± 6.0	28.3 ± 5.9	32.0 ± 6.3	0.002
Smoking (%)				0.69
Nonsmoker	48.0	48.4	44.9	
Ex-smoker	31.1	30.6	36.1	
Smoker	20.8	21.0	19.1	
Dietary supplements (% users)	62.4	62.5	62.0	0.95
Selenium (μg/L)	137.1 ± 19.9	136.4 ± 19.9	143.7 ± 18.3	0.001
Plasma glucose (mg/dL)	102.2 ± 23.9	96.9 ± 9.6	149.9 ± 48.9	—
Glycosylated hemoglobin (%)	5.6 (0.7)	5.4 (0.3)	7.0 (1.4)	—

Values are survey-weighted mean ± SD or percentage for continuous or categorical variables, respectively.

**Table 2:** Characteristics of the study population by serum selenium quartile (Q).

	Quartile of serum selenium				
Characteristic	Q1 (< 124 μg/L)	Q2 (124–133 μg/L)	Q3 (134–146 μg/L)	Q4 (≥ 147 μg/L)	*p*-Trend
No.	208	220	252	237	
Age (years)	52.8	54.6	54.5	54.8	0.001
Sex (% female)	61.7	59.7	47.4	47.0	0.07
Race (%)					
Non-Hispanic white	72.1	73.5	74.5	82.6	0.04
Non-Hispanic black	17.8	10.4	8.3	5.2	0.007
Mexican American	5.3	5.4	5.3	8.2	0.16
Other	4.8	10.8	11.9	4.0	0.30
Education (% high school)	80.9	83.3	84.9	85.5	0.37
BMI (kg/m^2^)	29.2	28.9	28.4	28.1	0.02
Smoking (%)					
Nonsmoker	51.6	47.1	48.2	45.4	0.32
Ex-smoker	18.0	32.5	32.8	39.8	0.006
Smoker	30.4	20.4	18.9	14.8	0.005
Dietary supplements (% users)	45.1	58.3	70.4	73.1	0.003
Selenium (μg/L)	115.7	128.6	139.2	161.8	—

Values are survey weighted mean or percentage for continuous or categorical variables, respectively.

**Table 3:** Adjusted ORs (95% CI) for the presence of diabetes and adjusted differences in fasting glucose and glycosylated hemoglobin comparing the three highest quartiles (Q) with the first quartile of serum selenium.

	Quartile of serum selenium				
Model	Q1 (< 124 μg/L)	Q2 (124–133 μg/L)	Q3 (134–146 μg/L)	Q4 (≥ 147 μg/L)	*p*-Trend
Diabetes (%)	3.6	10.0	9.6	16.1	
Model 1	1.00 (Reference)	2.91 (1.15 to 7.33)	2.70 (0.88 to 8.29)	5.24 (2.46 to 11.17)	0.01
Model 2	1.00 (Reference)	3.14 (1.00 to 9.87)	3.57 (1.25 to 10.17)	7.46 (3.32 to 16.75)	0.002
Model 3	1.00 (Reference)	3.18 (1.01 to 9.96)	3.65 (1.31 to 10.16)	7.64 (3.34 to 17.46)	0.002
Plasma glucose (mg/dL)	98.6	102.1	101.2	106.5	
Model 1	0.0 (Reference)	3.00 (− 0.09 to 6.09)	1.36 (− 2.43 to 5.15)	6.98 (0.57 to 13.39)	0.09
Model 2	0.0 (Reference)	3.43 (− 0.12 to 6.98)	2.76 (− 0.45 to 5.97)	9.04 (2.75 to 15.33)	0.01
Model 3	0.0 (Reference)	3.58 (0.05 to 7.12)	3.15 (0.15 to 6.14)	9.46 (3.35 to 15.56)	0.01
Model 4	0.0 (Reference)	4.17 (− 0.39 to 8.73)	3.98 (− 0.55 to 8.51)	10.73 (6.17 to 15.28)	< 0.001
Glycosylated hemoglobin (%)	5.47	5.55	5.52	5.68	
Model 1	0.0 (Reference)	0.07 (− 0.06 to 0.20)	0.05 (− 0.04 to 0.14)	0.23 (0.07 to 0.39)	0.06
Model 2	0.0 (Reference)	0.08 (− 0.07 to 0.24)	0.08 (0.00 to 0.16)	0.28 (0.11 to 0.44)	0.01
Model 3	0.0 (Reference)	0.09 (− 0.06 to 0.25)	0.10 (0.02 to 0.17)	0.30 (0.14 to 0.46)	0.007
Model 4	0.0 (Reference)	0.11 (− 0.03 to 0.24)	0.12 (− 0.01 to 0.25)	0.33 (0.19 to 0.46)	< 0.001

The first row for each outcome shows the unadjusted (survey-weighted) proportions (of diabetes) and averages (of plasma glucose and glycosylated hemoglobin). Models 1–3 used multiple linear or logistic regression models with survey weights, strata, and clusters to account for complex survey design. Model 4 used censored regression with survey weights only. For results related to the prevalence of diabetes, prevalence ORs are not equivalent to prevalence ratios. To illustrate this difference, the prevalence ratios for diabetes comparing quartiles 2–4 versus the first quartile for model 3, estimated using marginal standardization, were 2.73, 3.05, and 5.32, respectively.
